# Supplementary material for: Modeling glioblastoma heterogeneity as a dynamic network of cell states
Source: Mol Syst Biol. 2021 Sep 16;17(9):e10105. doi: 10.15252/msb.202010105 (PMC8444284; doi:10.15252/msb.202010105)
Supplement: Supplementary file 6 — Source Data for Figure 5 [file MSB-17-e10105-s004.zip › Figure5A_sourcedata/GSEA_3017/nosigdb_stateE.GseaPreranked.1622795031093/gsea_report_for_na_neg_1622795031093.html]

Report for na\_neg 1622795031093 [GSEA]

| GS  follow link to MSigDB | GS DETAILS | SIZE | ES | NES | NOM p-val | FDR q-val | FWER p-val | RANK AT MAX | LEADING EDGE || 1 | DOROSZKO\_U3013MG\_CLUSTER\_2 | Details ... | 180 | -0.74 | -4.28 | 0.000 | 0.000 | 0.000 | 201 | tags=70%, list=21%, signal=72% |
| 2 | LARSSON\_STATE\_4 | Details ... | 96 | -0.77 | -4.08 | 0.000 | 0.000 | 0.000 | 175 | tags=77%, list=18%, signal=85% |
| 3 | FELDMAN\_2020\_G1 | Details ... | 127 | -0.73 | -4.04 | 0.000 | 0.000 | 0.000 | 204 | tags=76%, list=22%, signal=84% |
| 4 | RICHARDS\_ZHONG\_NATURE\_2018\_NPCS\_UPREG | Details ... | 97 | -0.77 | -3.95 | 0.000 | 0.000 | 0.000 | 197 | tags=80%, list=21%, signal=91% |
| 5 | DOROSZKO\_U3054MG\_CLUSTER\_4 | Details ... | 326 | -0.66 | -3.94 | 0.000 | 0.000 | 0.000 | 374 | tags=75%, list=39%, signal=81% |
| 6 | DALMO\_BARCODENUMBER\_UNPUB | Details ... | 107 | -0.72 | -3.93 | 0.000 | 0.000 | 0.000 | 166 | tags=69%, list=18%, signal=74% |
| 7 | RICHARDS\_NOWAKOWSKI\_SCIENCE\_2017\_IPC\_DIV2\_UPREG | Details ... | 387 | -0.62 | -3.78 | 0.000 | 0.000 | 0.000 | 362 | tags=67%, list=38%, signal=64% |
| 8 | RICHARDS\_NOWAKOWSKI\_SCIENCE\_2017\_MGE\_DIV\_UPREG | Details ... | 326 | -0.63 | -3.76 | 0.000 | 0.000 | 0.000 | 304 | tags=63%, list=32%, signal=61% |
| 9 | DOROSZKO\_U3180MG\_CLUSTER\_3 | Details ... | 278 | -0.63 | -3.73 | 0.000 | 0.000 | 0.000 | 294 | tags=65%, list=31%, signal=66% |
| 10 | RICHARDS\_NOWAKOWSKI\_SCIENCE\_2017\_MGE\_IPC2\_UPREG | Details ... | 301 | -0.63 | -3.72 | 0.000 | 0.000 | 0.000 | 256 | tags=58%, list=27%, signal=54% |
| 11 | RICHARDS\_NOWAKOWSKI\_SCIENCE\_2017\_RG\_EARLY\_UPREG | Details ... | 134 | -0.67 | -3.67 | 0.000 | 0.000 | 0.000 | 163 | tags=58%, list=17%, signal=60% |
| 12 | RICHARDS\_NOWAKOWSKI\_SCIENCE\_2017\_IPC\_DIV1\_UPREG | Details ... | 329 | -0.61 | -3.64 | 0.000 | 0.000 | 0.000 | 276 | tags=58%, list=29%, signal=53% |
| 13 | GAROFANO\_IAVARONE\_PPR\_SUBTYPE | Details ... | 244 | -0.61 | -3.56 | 0.000 | 0.000 | 0.000 | 283 | tags=63%, list=30%, signal=67% |
| 14 | FELDMAN\_2020\_G2-M | Details ... | 180 | -0.62 | -3.56 | 0.000 | 0.000 | 0.000 | 204 | tags=61%, list=22%, signal=63% |
| 15 | FELDMAN\_2020\_S-G2 | Details ... | 212 | -0.60 | -3.50 | 0.000 | 0.000 | 0.000 | 190 | tags=53%, list=20%, signal=51% |
| 16 | RICHARDS\_NOWAKOWSKI\_SCIENCE\_2017\_MGE\_IPC1\_UPREG | Details ... | 266 | -0.59 | -3.49 | 0.000 | 0.000 | 0.000 | 287 | tags=61%, list=30%, signal=62% |
| 17 | NB\_KI\_CLUSTER5 | Details ... | 81 | -0.68 | -3.46 | 0.000 | 0.000 | 0.000 | 293 | tags=86%, list=31%, signal=114% |
| 18 | SEURAT\_CELLCYCLE | Details ... | 82 | -0.68 | -3.45 | 0.000 | 0.000 | 0.000 | 215 | tags=73%, list=23%, signal=86% |
| 19 | IVY\_CT | Details ... | 92 | -0.66 | -3.44 | 0.000 | 0.000 | 0.000 | 192 | tags=68%, list=20%, signal=78% |
| 20 | RICHARDS\_NEFTEL\_CELL\_2019\_G2\_M | Details ... | 40 | -0.69 | -3.07 | 0.000 | 0.000 | 0.000 | 250 | tags=85%, list=26%, signal=111% |
| 21 | SUVA\_G2M\_PMID\_31327527 |  | 40 | -0.69 | -3.04 | 0.000 | 0.000 | 0.000 | 250 | tags=85%, list=26%, signal=111% |
| 22 | RICHARDS\_NOWAKOWSKI\_SCIENCE\_2017\_U2\_UPREG |  | 407 | -0.50 | -3.00 | 0.000 | 0.000 | 0.000 | 264 | tags=47%, list=28%, signal=37% |
| 23 | FELDMAN\_2020\_NEURAL G0 |  | 154 | -0.53 | -2.99 | 0.000 | 0.000 | 0.000 | 203 | tags=53%, list=21%, signal=56% |
| 24 | RICHARDS\_NOWAKOWSKI\_SCIENCE\_2017\_RG\_DIV1\_UPREG |  | 491 | -0.49 | -2.95 | 0.000 | 0.000 | 0.000 | 345 | tags=53%, list=36%, signal=40% |
| 25 | GAROFANO\_IAVARONE\_LIST\_OF\_NEURAL\_PROGENITOR\_MARKER\_GENES |  | 52 | -0.61 | -2.88 | 0.000 | 0.000 | 0.000 | 264 | tags=75%, list=28%, signal=98% |
| 26 | FELDMAN\_2020\_S |  | 104 | -0.53 | -2.85 | 0.000 | 0.000 | 0.000 | 210 | tags=55%, list=22%, signal=63% |
| 27 | RICHARDS\_NOWAKOWSKI\_SCIENCE\_2017\_MGE\_RG2\_UPREG |  | 149 | -0.48 | -2.73 | 0.000 | 0.000 | 0.000 | 263 | tags=54%, list=28%, signal=63% |
| 28 | RICHARDS\_NEFTEL\_CELL\_2019\_G1\_S |  | 26 | -0.69 | -2.72 | 0.000 | 0.000 | 0.000 | 201 | tags=77%, list=21%, signal=95% |
| 29 | SUVA\_G1S\_PMID\_31327527 |  | 26 | -0.69 | -2.72 | 0.000 | 0.000 | 0.000 | 201 | tags=77%, list=21%, signal=95% |
| 30 | RICHARDS\_CAHOY\_JNEUROSCI\_2010\_ASTRO\_YOUNG |  | 200 | -0.44 | -2.55 | 0.000 | 0.000 | 0.000 | 189 | tags=41%, list=20%, signal=40% |
| 31 | FELDMAN\_2020\_LATEG1 |  | 56 | -0.52 | -2.51 | 0.000 | 0.000 | 0.000 | 83 | tags=41%, list=9%, signal=42% |
| 32 | DOROSZKO\_U3180MG\_CLUSTER\_4 |  | 61 | -0.49 | -2.38 | 0.000 | 0.000 | 0.001 | 324 | tags=69%, list=34%, signal=98% |
| 33 | RICHARDS\_NOWAKOWSKI\_SCIENCE\_2017\_RG\_DIV2\_UPREG |  | 368 | -0.39 | -2.34 | 0.000 | 0.000 | 0.004 | 264 | tags=42%, list=28%, signal=35% |
| 34 | SINGH\_SOX2\_UPREGULATED\_TRANSCRIPTS\_PMID\_28122245 |  | 87 | -0.43 | -2.23 | 0.000 | 0.000 | 0.005 | 295 | tags=54%, list=31%, signal=71% |
| 35 | FELDMAN\_2020\_M-EARLYG1 |  | 56 | -0.46 | -2.19 | 0.001 | 0.000 | 0.011 | 174 | tags=46%, list=18%, signal=54% |
| 36 | PARADA\_T1\_UP\_26461091 |  | 31 | -0.53 | -2.15 | 0.000 | 0.000 | 0.017 | 132 | tags=52%, list=14%, signal=58% |
| 37 | DOROSZKO\_U3054MG\_CLUSTER\_6 |  | 57 | -0.42 | -2.03 | 0.000 | 0.001 | 0.080 | 324 | tags=63%, list=34%, signal=90% |
| 38 | PARADA\_T2\_UP\_26461091 |  | 28 | -0.51 | -2.01 | 0.000 | 0.001 | 0.099 | 202 | tags=57%, list=21%, signal=70% |
| 39 | RICHARDS\_CAHOY\_JNEUROSCI\_2016\_CULTURED\_ASTROGLIA |  | 238 | -0.34 | -1.97 | 0.000 | 0.002 | 0.135 | 173 | tags=32%, list=18%, signal=30% |
| 40 | VANGRONINGEN\_ADRN |  | 65 | -0.39 | -1.94 | 0.000 | 0.002 | 0.177 | 197 | tags=40%, list=21%, signal=47% |
| 41 | RICHARDS\_NOWAKOWSKI\_SCIENCE\_2017\_MGE\_IPC3\_UPREG |  | 36 | -0.42 | -1.85 | 0.009 | 0.005 | 0.352 | 203 | tags=44%, list=21%, signal=54% |
| 42 | OLSEN\_ADRENERGIC |  | 22 | -0.45 | -1.68 | 0.024 | 0.020 | 0.816 | 203 | tags=41%, list=21%, signal=51% |
| 43 | LARSSON\_STATE\_3 |  | 20 | -0.46 | -1.67 | 0.025 | 0.021 | 0.834 | 154 | tags=40%, list=16%, signal=47% |
| 44 | RICHARDS\_CAHOY\_JNEUROSCI\_2012\_OPC |  | 349 | -0.28 | -1.66 | 0.001 | 0.021 | 0.847 | 191 | tags=28%, list=20%, signal=22% |
| 45 | RICHARDS\_NEFTEL\_CELL\_2019\_NPC1 |  | 17 | -0.46 | -1.59 | 0.051 | 0.036 | 0.952 | 202 | tags=53%, list=21%, signal=66% |
| 46 | SUVA\_NPC1\_PMID\_31327527 |  | 17 | -0.46 | -1.55 | 0.045 | 0.046 | 0.987 | 202 | tags=53%, list=21%, signal=66% |
| 47 | FELDMAN\_2020\_LOWRNA |  | 194 | -0.25 | -1.45 | 0.027 | 0.079 | 0.998 | 182 | tags=26%, list=19%, signal=25% |
| 48 | KLING\_GBM\_PHENO\_PMID\_27667176 |  | 28 | -0.29 | -1.19 | 0.272 | 0.306 | 1.000 | 191 | tags=36%, list=20%, signal=43% |
| 49 | NB\_KI\_CLUSTER3 |  | 29 | -0.29 | -1.18 | 0.257 | 0.315 | 1.000 | 658 | tags=97%, list=69%, signal=307% |
| 50 | TF\_CENSUS\_TAIPALE |  | 87 | -0.23 | -1.18 | 0.254 | 0.310 | 1.000 | 321 | tags=39%, list=34%, signal=54% |
| 51 | RICHARDS\_NOWAKOWSKI\_SCIENCE\_2017\_NIN1\_UPREG |  | 20 | -0.31 | -1.15 | 0.306 | 0.338 | 1.000 | 232 | tags=45%, list=24%, signal=58% |
| 52 | ALMSTEDT\_17Q\_MSIGDB |  | 27 | -0.29 | -1.15 | 0.291 | 0.332 | 1.000 | 219 | tags=41%, list=23%, signal=51% |
| 53 | RICHARDS\_VERHAAK\_CANCERCELL\_2010\_PRONEURAL |  | 32 | -0.25 | -1.06 | 0.432 | 0.463 | 1.000 | 272 | tags=44%, list=29%, signal=59% |
| 54 | NB\_KI\_CLUSTER7 |  | 23 | -0.27 | -1.04 | 0.422 | 0.482 | 1.000 | 697 | tags=100%, list=74%, signal=370% |
| 55 | RICHARDS\_NOWAKOWSKI\_SCIENCE\_2017\_NIN4\_UPREG |  | 19 | -0.24 | -0.89 | 0.595 | 0.693 | 1.000 | 211 | tags=37%, list=22%, signal=46% |
| 56 | RICHARDS\_NOWAKOWSKI\_SCIENCE\_2017\_U3\_UPREG |  | 86 | -0.16 | -0.82 | 0.717 | 0.784 | 1.000 | 254 | tags=29%, list=27%, signal=36% |
| 57 | KRONA\_ROSEN\_WHITE\_MATTER\_INVASION |  | 36 | -0.18 | -0.78 | 0.722 | 0.824 | 1.000 | 213 | tags=28%, list=22%, signal=34% |
| 58 | ALMSTEDT\_11Q\_MSIGDB |  | 34 | -0.17 | -0.71 | 0.839 | 0.893 | 1.000 | 159 | tags=21%, list=17%, signal=24% |
| 59 | RICHARDS\_INHOUSE\_BULKRNASEQ\_2019\_DEVELOPMENTALGSC |  | 161 | -0.12 | -0.68 | 0.928 | 0.909 | 1.000 | 540 | tags=63%, list=57%, signal=122% |
| 60 | RICHARDS\_NOWAKOWSKI\_SCIENCE\_2017\_IPC\_NEN1\_UPREG\_1 |  | 20 | -0.17 | -0.65 | 0.862 | 0.919 | 1.000 | 184 | tags=25%, list=19%, signal=30% |
| 61 | NB\_KI\_CLUSTER8 |  | 29 | -0.15 | -0.63 | 0.888 | 0.914 | 1.000 | 647 | tags=83%, list=68%, signal=253% |
Table: Gene sets enriched in phenotype **na **[plain text format]****
